# Antibiotic Resistance among *Fusobacterium, Capnocytophaga*, and *Leptotrichia* Species of the Oral Cavity

**DOI:** 10.3290/j.ohpd.b4009553

**Published:** 2023-04-04

**Authors:** Thomas Thurnheer, Sabrina Bensland, Sigrun Eick, Eva M. Kulik, Thomas Attin, Lamprini Karygianni

**Affiliations:** a Senior Scientist, Clinic of Conservative and Preventive Dentistry, Center of Dental Medicine, University of Zürich, Switzerland. Idea, conceptualisation, validation, wrote and proofread the manuscript.; b Dentist, Clinic of Conservative and Preventive Dentistry, Center of Dental Medicine, University of Zürich, Switzerland. Experimental design, collected data, performed laboratory tests, original draft preparation.; c Associate Professor, Department of Periodontology, School of Dental Medicine, University of Bern, Bern, Switzerland. Validation, contributed substantially to the discussion, reviewed the manuscript.; d Senior Scientist, Department Research, University Center for Dental Medicine UZB, University of Basel, Basel, Switzerland. Reviewed the manuscript, contributed substantially to the discussion.; e Professor and Chair, Clinic of Conservative and Preventive Dentistry, Center of Dental Medicine, University of Zürich, Switzerland. Experimental design, validation, original draft preparation, reviewed the manuscript.; f Dentist, Clinic of Conservative and Preventive Dentistry, Center of Dental Medicine, University of Zürich, Switzerland. Idea, hypothesis, conceptualisation, validation, wrote and reviewed the manuscript.

**Keywords:** antibiotics, resistance, oral bacteria, clinical isolates, E-test

## Abstract

**Purpose:**

Antibiotics play an important role in treating periodontal diseases. Due to the effectiveness of antibiotic therapies, their usage in dentistry has significantly increased. The aim of this study focused on the in-vitro susceptibility of different gram-negative oral bacteria species – which are associated with periodontal diseases (*Fusobacterium* spp., *Capnocytophaga* spp. and *Leptotrichia buccalis*) and have different geographical origins (Asia and Europe) – against antimicrobials that are clinically relevant in dental therapy.

**Materials and Methods:**

A total of 45 strains were tested (29 *Fusobacterium* spp., 13 *Capnocytophaga* spp. and 3 *L. buccalis*) that were either isolated from Chinese patients or were obtained from different strain collections. Their antimicrobial susceptibility to the antimicrobial agents benzylpenicillin, amoxicillin, amoxicillin-clavulanic acid, ciprofloxacin, moxifloxacin, clindamycin, doxycycline, tetracycline and metronidazole was tested using the E-Test. Strains with particular resistance to penicillin, clindamycin and metronidazole were further analysed for resistance genes.

**Results:**

All tested bacterial isolates were sensitive to amoxicillin, amoxicillin-clavulanic acid, doxycycline and tetracycline, but showed variable sensitivity towards other antibiotics such as benzylpenicillin, ciprofloxacin, moxifloxacin, clindamycin and metronidazole.

**Conclusion:**

The results of the present study suggest that certain periodontal disease-related bacterial strains can be resistant towards antimicrobial agents commonly used in adjuvant periodontal therapy.

The balance, stability and composition of the oral microbiota are crucial for maintaining oral health and preventing the development of periodontal diseases such as gingivitis and periodontitis. These diseases are initiated by an accumulation of microbial biofilms, resulting in the loss of symbiosis between the microorganisms in the biofilm and the host immune response.^43^ Clinical signs of oral biofilm-induced gingivitis include redness and swelling of the keratinised gingiva and bleeding on probing caused by the inflamed gum tissue.^36^ Gram-positive bacteria such as *Streptococcus* and *Actinomyces* species dominate in a healthy gingival sulcus, whereas in gingival sites with clinical signs of gingivitis an increased number of gram-negative bacteria such as *Fusobacterium nucleatum*, *Leptotrichia* spp. and *Tannerella* spp. are found.^49,61^

Gingivitis is a reversible disease, but if left untreated, it may develop into periodontitis. This oral disease results from dysbiosis due to a breakdown in host-microbe homeostasis causing inflammation and destruction of the periodontal tissue that can lead to tooth loss if not treated properly.^17,53^ Many factors such as immune deficiencies, smoking and stress have been shown to influence the severity of periodontal diseases.^3,51,81^ Periodontitis is one of the most prevalent oral diseases globally,^50^ and it is associated with higher counts of several genera, e.g. *Fusbacterium, Porphyromonas, Leptotrichia, Tannerella* and *Treponema*.^45^

According to the current classification of periodontal disease, two other forms are listed in addition to periodontitis: periodontitis as a manifestation of a systemic disease and necrotising periodontal diseases, i.e. necrotising gingivitis, necrotising periodontitis and necrotising stomatitis.^14^ Necrotising gingivitis (NG) has a prevalence of less than 1% and therefore qualifies as a rare disease. The mean age of patients with NG ranges from 20 to 24 years and seems to be more common in malnourished children and young adults as well as in patients with immunodeficiency. Other risk factors include high-stress levels and smoking.^7,19^ NG differs from other periodontal diseases in its severe clinical course with acute and rapidly progressing destruction of gingiva resulting in strong pain, necrosis and bleeding of the interdental papilla, foetor ex ore as well as signs of fever, regional lymphadenopathy and malaise. Bacteria mainly associated with NG are *Fusobacterium* spp. and spirochetes such as *Treponema* spp.^7,19^

In both severe periodontitis and severe necrotising periodontal diseases, antibiotics are often applied.^1,20,23,60,75^ Adjunctive use of amoxicillin and metronidazole in non-surgical periodontal therapy has a positive effect on clinical outcomes.^67^ Patients with NG are usually administered metronidazole, which is considered to be effective against anaerobic microorganisms.^19,62^ With respect to anaerobic oral microorganisms, the antibiotic resistance pattern of *Porphyromonas gingivalis* has been analysed frequently and widely from different geographical regions.^28,41,73^ However, data regarding *F. nucleatum, Leptotrichia* spp. and *Capnocytophaga* spp. are rare. *F. nucleatum* belongs to the genus *Fusobacterium* and is an anaerobic, gram-negative bacterium.^9^ Although *F. nucleatum* can also be detected in healthy patients, it is more likely to be found in diseased sites.^9^

The genus *Capnocytophaga* includes gram-negative, anaerobic bacteria.^39^ The species *C. gingivalis, C. granulosa, C. haemolytica, C. leadbetteri, C. ochracea* and *C. sputigena* can be found in the healthy human oral microflora.^37^ However, *Capnocytophaga* spp. are also regularly isolated from patients with mucosal ulcerations, bleeding gums and gingivitis, as well as from immunocompromised patients with systemic infections.^38^ Both *F. nucleatum* and *Capnocytophaga* spp. are described as pathogenic because of their ability to destroy surrounding tissue and weaken the defense mechanisms of the host, which is attributed to protease enzymes.^24^

*Leptotrichia* species are anaerobic, gram-negative bacteria belonging to the family Leptotrichiaceae and are described as opportunistic pathogens. *Leptotrichia* spp. are non-motile bacteria, which ferment carbohydrates to produce various organic acids.^26^ They are found in higher quantities in NG^49^ and periodontitis.^45^ Interestingly, recent studies showed a correlation between *Leptotrichia* spp. and rheumatoid arthritis,^58^ bacteremia and cancer.^68,76^

Antimicrobial resistance (AMR) is considered by all major regulatory, economic and political bodies to be one of the major global health challenges of the 21st century and should therefore remain an ongoing research topic.^8,35^ As the prevalence of resistance varies between different geographic locations,^72^ susceptibility profiles of oral bacteria should be known in order to ensure the most effective antibiotic therapy for patients.^11^ Therefore, the aim of this study was to determine the in-vitro susceptibility of different gram-negative oral bacteria species – which are associated with periodontal diseases (*Fusobacterium* spp., *Capnocytophaga* spp. and *L. buccalis*) and have different geographical origins (Asia and Europe) – to antimicrobials that are clinically relevant in dental therapy.

## Materials and Methods

### Bacterial Strains

The study protocol was approved by the Ethics Committee of the University of Zürich (Basec Nr. Req-2019-01260). While conducting data sampling and the subsequent evaluation, relevant institutional and national guidelines/regulations were followed at all times. The samples were collected from a total of 42 healthy Chinese patients (26 to 53 years of age) from dental clinics in Beidaihe, Chengede, Shijiazhuang and Xi’an in China. All of them gave their written informed consent.^32^ From each subject, a total of three plaque samples were collected from the most disease-affected gingival surfaces, which had to be located in two different quadrants. Following a procedure previously described by the present and other authors,^5,31,32,77,78,82,83^ sample material from three sites was pooled in 1 ml of reduced transport fluid containing 10% glycerol.^44^ The samples were then aliquoted and stored in liquid nitrogen (N_2_) until use.

The strains of *Fusobacterium* spp., *Capnocytophaga* spp. and *L. buccalis* used in this study were either isolated from the clinical samples described above or were obtained from different strain collections (Table 1). Identification and characterisation of the isolated bacterial strains has been described previously^30-32^ based on immunofluorescence identification tests with well-characterised monoclonal antibodies, fluorescence in-situ hybridisation assay probes with specific 16S rRNA targeted probes for the different fusiform genera, and 16S rDNA sequence analysis. Additionally, *F. nucleatum* OMZ 772 was identified using PCR^29^ and *Leptotrichia* strains were additionally verified by MALDI-TOF MS (microflex LT, Bruker Daltonics; Bremen, Germany).

**Table 1:** Strains used in this study, origin and associated disease

Genus	Species	Isolate No.^a^	Origin^b^	Disease^c^
*Fusobacterium*	*Fusobacterium sp.*	OMZ 981	China^d^	Gingivitis
	*Fusobacterium sp.*	OMZ 982	China^d^	NG
	*Fusobacterium sp.*	OMZ 986	China^d^	NG
	*Fusobacterium sp.*	OMZ 989	China^d^	NG
	*F. nucleatum*	17AF1	China^d^	Gingivitis
	*F. nucleatum*	20AF1	China^d^	Gingivitis
	*F. nucleatum*	38AF2	China^d^	NG
	*F. nucleatum*	42AF2	China^d^	NG
	*F. nucleatum*	43AF2	China^d^	NG
	*F. nucleatum*	45AF2	China^d^	NG
	*F. nucleatum*	47AF2	China^d^	NG
	*F. nucleatum*	48AF1	China^d^	NG
	*F. nucleatum*	OMZ 274	Switzerland^e^	Not further specified
	*F. nucleatum*	OMZ 373	FDC^e^	Not further specified
	*F. nucleatum*	OMZ 439	Switzerland^e^	Not further specified
	*F. nucleatum*	OMZ 567	Switzerland^e^	Not further specified
	*F. nucleatum*	OMZ 643	Sweden^e^	Not further specified
	*F. nucleatum*	OMZ 647	Sweden^e^	Not further specified
	*F. nucleatum*	OMZ 648	Sweden^e^	Not further specified
	*F. nucleatum*	OMZ 760	Switzerland^e^	Not further specified
	*F. nucleatum*	OMZ 772	Switzerland^e^	Periodontitis
	*F. nucleatum*	OMZ 773	Switzerland^e^	Periodontitis
	*F. nucleatum*	OMZ 776	Switzerland^e^	Periodontitis
	*F. nucleatum*	OMZ 818	Switzerland^e^	Not further specified
	*F. nucleatum ssp. animalis*	OMZ 990	China^d^	Gingivitis
	*F. nucleatum ssp. nucleatum*	OMZ 598	Germany^e^	Not further specified
	*F. periodonticum*	OMZ 599	Germany^e^	Not further specified
	*F. periodonticum*	OMZ 636	ATCC^e^	Periodontitis
	*F. periodonticum*	OMZ 988	China^d^	NG
*Capnocytophaga*	*Capnocytophaga sp.*	19AF3	China^d^	Gingivitis
	*Capnocytophaga sp.*	21AK1	China^d^	Gingivitis
	*Capnocytophaga sp.*	47AK1	China^d^	NG
	*Capnocytophaga sp.*	7AF2	China^d^	Gingivitis
	*Capnocytophaga sp.*	8AK1	China^d^	Gingivitis
	*Capnocytophaga sp.*	OMZ 290	Switzerland^e^	Not further specified
	*Capnocytophaga sp.*	OMZ 291	Switzerland^e^	Not further specified
	*Capnocytophaga sp.*	OMZ 686	Switzerland^e^	Not further specified
	*Capnocytophaga gingivalis*	OMZ 435	ATCC^e^	Periodontitis
	*Capnocytophaga gingivalis*	OMZ 574	Switzerland^e^	Not further specified
	*Capnocytophaga ochracea*	OMZ 362	The Netherlands^e^	Not further specified
	*Capnocytophaga ochracea*	OMZ 436	ATCC^e^	Not further specified
	*Capnocytophaga sputigena*	OMZ 437	ATCC^e^	Periodontitis
*Leptotrichia*	*Leptotrichia buccalis*	7AF1	China^d^	Gingivitis
	*Leptotrichia buccalis*	OMZ 531	ATCC^e^	Not further specified
	*Leptotrichia buccalis*	OMZ 577	Switzerland^e^	Not further specified

^a^OMZ, Oral Microbiology, Zürich, Switzerland; ^b^ATCC (American Type Culture Collection); FDC: Forsyth Dental Center, Boston, MA, USA; ^c^NG: necrotising gingivitis; ^d^clinical isolate; ^e^laboratory strain.

All bacterial strains were cultured on Columbia Blood Agar (CBA) (Difco Columbia Blood Agar Base, BD; Franklin Lakes, NJ, USA) plates. *L. buccalis* and *Fusobacterium* spp. strains were incubated anaerobically (5% CO_2_, 10% H_2_, 85% N_2_), while *Capnocytophaga* strains grew aerobically with 10% CO_2_ at 37°C for 48 h.

### Antimicrobial Susceptibility Testing using the E-Test

Antimicrobial susceptibilities were determined using the E-test (BioMérieux, Marcy-1 Etoile; Craponne, France). The following nine antimicrobial agents were tested: benzylpenicillin, amoxicillin, amoxicillin-clavulanic acid, ciprofloxacin, moxifloxacin, clindamycin, doxycycline, tetracycline and metronidazole. Colonies of *Capnocytophaga, Fusobacterium* and *L. buccalis* strains were suspended in 0.9% NaCl solution and spread on Brucella blood agar plates before E-Test strips were placed on the agar plates.

The plates were then incubated under appropriate conditions (aerobically with 10% CO_2_ at 37°C for 24 h for *Capnocytophaga* strains, anaerobically at 37°C for 48 h for *Fusobacterium* spp. and *L. buccalis*). As metronidazole needs anaerobic incubation, it was not tested with the *Capnocytophaga* strains. The MICs (minimum inibitory concentrations) were determined according to the manufacturer’s instructions. The breakpoints were set according to the European Committee on Antimicrobial Susceptibility Testing (EUCAST) guidelines.^27^ In case of missing species, the clinical breakpoints of the closest relatives were chosen.

### Identification of Selected Resistance Genes

Strains with a particular resistance were sent to the Department of Periodontology lab at the University of Bern for the analysis of resistance genes. In the case of penicillin resistance, nitrocefin disks (Oxoid; Basingstoke, UK) were used to screen for beta-lactamases. Given clindamycin resistance, PCR for cfxA and ermF genes was performed, and where unusual metronidazole-resistant strains were found, these were screened for the nim gene. The primers were made according to the literature.^69,70^ To find mutations in QRDR of GyrA in *Leptotrichia* spp., primers (fwd.: 5’-ACT GAC ACA TCT TTT ACG CTC G-3’; rev. 5’-ACG ATG CAC GGA TTT TGA CA-3’) were designed according to a database (#NC_013192.1) by using Primer-BLAST. The PCR amplificate was sent to the Department of Periodontology lab at the University of Bern for sequencing analysis.

## Results

Of the included 45 strains, 19 were isolated in Europe (Switzerland, Sweden and Germany), 20 in China, and 6 originated from international strain collections. Among all tested antibiotics, no statistically significant differences were found in the MIC values between strains from China and Europe (p > 0.05, Mann-Whitney test).

### Resistance against Diverse Antibiotics was Detected for All Tested Clinical Isolates

**Fig 1:**
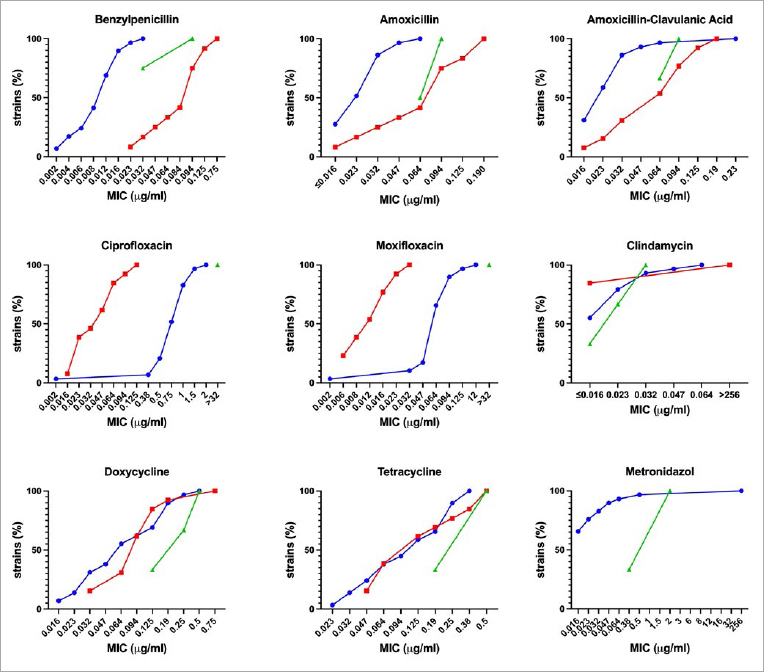
Cumulative minimal inhibitory concentrations against isolates of *Fusobacterium* spp. (blue), *Capnocytophaga* spp. (red) and *Leptotrichia buccalis* (green). Results are shown for the antibiotics benzylpenicillin, amoxicillin, amoxicillin-clavulanic acid, ciprofloxacin moxifloxacin, clindamycin, doxycycline, tetracycline and metronidazole.

Table 2 lists the obtained MICs with resistant bacteria marked in bold. Figure 1 shows the cumulative MICs against *Fusobacterium* spp., *Capnocytophaga* spp., and *L. buccalis* isolates. For all bacterial species, i.e. *Fusobacterium* spp., *Capnocytophaga* spp., and *L. buccalis*, resistant isolates against benzylpenicillin, ciprofloxacin, moxifloxacin, clindamycin and metronidazole according to EUCAST criteria were found.

**Table 2:** In-vitro susceptibilities of the oral bacterial strains, resistant bacteria and their corresponding MICs are marked in bold

Species	Isolate No.	Benzylpenicillin	Amoxicillin	Amoxicillin-clavulanic acid	Ciprofloxacin	Moxifloxacin	Clindamycin	Doxycycline	Tetracycline	Metronidazole^a^
*Fusobacterium sp.*	OMZ 981	0.012	0.032	0.023	0.5	0.047	< 0.016	0.094	0.19	< 0.016
*Fusobacterium sp.*	OMZ 982	0.016	0.032	0.032	0.75	0.064	< 0.016	0.19	0.25	0.023
*Fusobacterium sp.*	OMZ 986	0.008	0.016	< 0.016	0.75	0.094	< 0.016	0.25	0.38	0.032
*Fusobacterium sp.*	OMZ 989	0.008	0.032	0.032	0.5	0.064	0.023	0.125	0.125	< 0.016
*F. nucleatum*	17AF1	0.002	0.016	< 0.016	< 0.002	< 0.002	< 0.016	< 0.016	0.032	< 0.016
*F. nucleatum*	20AF1	0.002	< 0.016	< 0.016	1	0.047	< 0.016	0.094	0.25	< 0.016
*F. nucleatum*	38AF2	0.006	0.032	0.032	0.75	0.064	0.016	0.25	0.38	0.016
***F. nucleatum***	42AF2	0.016	0.023	0.023	0.75	**12**	0.032	0.19	0.25	0.032
*F. nucleatum*	43AF2	0.012	0.016	0.032	1.5	0.125	0.032	0.19	0.25	< 0.016
*F. nucleatum*	45AF2	0.008	0.016	0.016	1	0.064	0.023	0.032	0.023	< 0.016
*F. nucleatum*	47AF2	0.016	0.047	0.047	1.5	0.094	0.016	0.064	0.125	0.064
*F. nucleatum*	48AF1	0.004	0.016	< 0.016	0.75	0.064	< 0.016	0.023	0.032	< 0.016
*F. nucleatum*	OMZ 274	0.023	0.064	0.032	1.5	0.125	0.064	0.5	0.25	< 0.016
*F. nucleatum*	OMZ 373	0.016	0.023	0.023	1	0.094	< 0.016	0.19	0.25	< 0.016
*F. nucleatum*	OMZ 439	0.012	0.032	0.032	1	0.064	0.023	0.032	0.064	< 0.016
*F. nucleatum*	OMZ 567	0.008	0.023	0.016	1	0.064	< 0.016	0.032	0.047	< 0.016
*F. nucleatum*	OMZ 643	0.023	0.047	0.047	0.5	0.064	0.032	0.064	0.094	< 0.016
*F. nucleatum*	OMZ 647	0.004	0.016	< 0.016	0.5	0.032	0.023	0.032	0.064	< 0.016
*F. nucleatum*	OMZ 648	0.008	0.023	0.032	0.38	0.032	0.016	0.016	0.032	< 0.016
*F. nucleatum*	OMZ 760	0.012	0.032	0.023	1	0.064	0.016	0.032	0.047	0.047
***F. nucleatum***	OMZ 772	0.012	0.032	0.023	1	0.064	0.047	0.064	0.125	**> 256**
*F. nucleatum*	OMZ 773	0.016	0.023	0.023	1.5	0.094	0.023	0.064	0.125	< 0.016
*F. nucleatum*	OMZ 776	0.012	0.032	0.032	2	0.064	0.023	0.047	0.064	< 0.016
*F. nucleatum*	OMZ 818	0.004	0.016	< 0.016	1	0.064	< 0.016	0.125	0.19	< 0.016
*F. nucleatum ssp. animalis*	OMZ 990	0.032	0.047	0.064	0.75	0.094	0.016	0.047	0.064	0.023
*F. nucleatum ssp. nucleatum*	OMZ 598	0.016	0.032	0.023	0.75	0.094	0.032	0.064	0.094	< 0.016
*F. periodonticum*	OMZ 599	0.006	0.023	< 0.016	0.75	0.064	0.016	0.19	0.38	0.023
*F. periodonticum*	OMZ 636	0.012	0.032	0.023	1	0.064	0.016	0.023	0.047	0.047
*F. periodonticum*	OMZ 988	0.012	0.023	0.023	0.75	0.094	0.023	0.19	0.25	0.5
***Capnocytophaga sp.***	19AF3	0.094	0.064	0.094	0.125	0.032	< 0.016	0.75	0.75	ND
***Capnocytophaga sp.***	21AK1	0.125	0.094	0.064	0.047	0.016	**> 256**	0.125	0.75	ND
*Capnocytophaga sp.*	47AK1	0.094	0.094	0.016	0.064	0.023	< 0.016	0.125	0.125	ND
***Capnocytophaga sp.***	7AF2	0.75	0.19	0.125	0.094	0.016	**> 256**	0.19	0.5	ND
*Capnocytophaga sp.*	8AK1	0.094	0.032	0.125	0.023	0.016	< 0.016	0.064	0.064	ND
*Capnocytophaga sp.*	OMZ 290	0.094	0.19	0.094	0.047	0.06	< 0.016	0.094	0.125	ND
*Capnocytophaga sp.*	OMZ 291	0.064	0.023	0.032	0.064	0.012	< 0.016	0.125	0.25	ND
*Capnocytophaga sp.*	OMZ 686	0.023	< 0.016	0.032	0.023	0.008	< 0.016	0.094	0.19	ND
*C. gingivalis*	OMZ 435	0.125	0.125	0.19	0.032	0.008	0.016	0.032	0.047	ND
*C. gingivalis*	OMZ 574	0.047	0.047	0.023	0.023	0.006	0.016	0.094	0.064	ND
*C. ochracea*	OMZ 362	0.094	0.064	0.064	0.064	0.023	< 0.016	0.094	0.125	ND
*C. ochracea*	OMZ 436	0.032	0.094	0.064	0.016	0.012	< 0.016	0.064	0.064	ND
*C. sputigena*	OMZ 437	0.094	0.094	0.094	0.023	0.006	< 0.016	0.032	0.047	ND
***L. buccalis***	7AF1	0.032	0.094	0.064	**> 32**	**> 32**	0.016	0.125	0.19	2
***L. buccalis***	OMZ 531	0.032	0.064	0.064	**> 32**	**> 32**	0.032	0.5	0.5	2
***L. buccalis***	OMZ 577	0.032	0.094	0.094	**> 32**	**> 32**	0.023	0.25	0.5	0.38
*Fusobacterium sp.*	OMZ 981	0.012	0.032	0.023	0.5	0.047	< 0.016	0.094	0.19	< 0.016
***L. buccalis***	OMZ 577	0.032	0.094	0.094	**> 32**	**> 32**	0.023	0.25	0.5	0.38

^a^ND: not determined.

#### Resistance against moxifloxacin and metronidazole was found for two F. nucleatum isolates

All isolates were highly susceptible to amoxicillin, amoxicillin-clavulanic acid, doxycycline and tetracycline. *Fusobacterium* isolates were also sensitive to benzylpenicillin and clindamycin, whereas two isolates, *F. nucleatum* 42AF. and *F. nucleatum* OMZ 772, were resistant to moxifloxacin and metronidazole, respectively (Table 2).

The highest MIC for metronidazole of all tested bacteria, with a value exceeding 256 µg/l, was found for *F. nucleatum* OMZ 772 (Table 2).

#### Resistance against ciprofloxacin and moxifloxacin was detected for three Leptotrichia isolates

The three *Leptotrichia* isolates were resistant to ciprofloxacin and moxifloxacin, but they were found to be sensitive to all other tested antibiotics (Table 2).

#### Resistance against clindamycin was detected for two Capnocytophaga isolates

*Capnocytophaga* isolates were sensitive to ciprofloxacin and moxifloxacin. Metronidazole was not tested with the *Capnocytophaga* isolates because this antibiotic needs anaerobic incubation. Two *Capnocytophaga* isolates were resistant to clindamycin, and the MIC of benzylpenicillin was high against one strain.

### Basis of Resistance

Selected strains were screened for the basis of resistance. No betalactamase activity of the strain *Capnocytophaga* 7AF. was found using nitrocefin disks. The nim gene was not identified in either *F. nucleatum* OMZ 772 or in the *Capnocytophaga* ssp. 19AF3 and 21AK1. Both *Capnocytophaga* ssp. 7AF2 and 21AK1 harbored the CfxA gene, the strain 7AF2, and also the ermF gene.

The *F. nucleatum* 42AF2 could no longer be cultured. Thus, only the three *Leptotrichia* ssp. were sent to the Department of Periodontology lab at the University of Bern for PCR of the QRDR of gyrA and respective sequencing. In two strains (OMZ 577 and 7AF1), a substitution of the nucleic acid G by A was found at position 2,456,868, but it did not result in an amino acid substitution.

## Discussion

Due to the effectiveness of antibiotic therapies, the usage of antibiotics in dentistry has increased. Consequently, oral bacteria with increased antibiotic resistance have emerged.^52^ Earlier studies concluded that it is important to improve the education of dental personnel on antibiotic use to prevent further development of antibiotic resistance.^65^ The current guidelines of the European Federation of Periodontology recommend the use of adjuvant antibiotics only for very select patient groups (young age, generalised periodontitis stage III) in cause-related therapy.^57^ The study by Van Winkelhoff et al^72^ showed that uncontrolled and increased prescription of systemic antibiotics can lead to increased microbial resistances; for instance, bacterial samples from Spain had an increased resistance to antibiotics compared to bacterial samples from the Netherlands, where antibiotics are prescribed less frequently. Anaerobic bacteria show different degrees of in-vitro susceptibility against antibiotics such as amoxicillin-clavulanic acid, clindamycin and metronidazole. In vitro, *Fusobacterium* spp. show high susceptibility to amoxicillin-clavulanic acid, metronidazole and moxifloxacin, whereas the susceptibility to amoxicillin and clindamycin varies.^38^ In this study, the E-test for *Fusobacterium* yielded 100% sensitivity for amoxicillin, amoxicillin-clavulanic acid and clindamycin. Only one *Fusobacterium* isolate was resistant to moxifloxacin or metronidazole. The fact that some anaerobic isolates were less susceptible to some antibiotics agrees with previous literature.^6,48^

Amoxicillin and amoxicillin-clavulanic acid were effective against all tested anaerobic bacterial species. This finding is supported by Kuriyama et al,^42^ who found high levels of activity of amoxicillin against anaerobic bacteria collected from dentoalveolar infections. Veloo et al^73^ found all tested oral pathogens, i.e. *Prevotella intermedia*/*P. nigrescens*, *F. nucleatum, P. gingivalis, Parvimonas micra* and *Aggregatibacter* a*ctinomycetemcomitans* to be susceptible to amoxicillin-clavulanic acid. For this reason, amoxicillin and amoxicillin-clavulanic acid appear to be good therapeutic agents against anaerobic oral bacteria.

All bacterial isolates proved to be sensitive against tetracycline. Tetracycline is active against various microorganisms, ranging from gram-positive and gram-negative bacteria to protozoans.^15^ Due to the high use of tetracyclines, susceptibility has decreased significantly over the years since its introduction in the 1950s.^54^ Resistance emerges primarily through new gene acquisition, which alter the efflux mechanism or the ribosomal protection.^65^ Less frequently, adaptation occurs by evolutionary mechanisms such as gene mutations.^15^ Villedieu et al^74^ was able to identify tetracycline resistant gram-positive and gram-negative bacteria of the oral cavity in periodontally healthy patients, with tetracycline-resistant bacteria constituting an average of 11% of the total cultivable oral microflora.

As stated previously, metronidazole has thus become a widely used systemic treatment option for periodontal diseases.^18^ The in-vitro susceptibilities determined in our study showed MICs exceeding 4 µg/l for *Capnocytophaga* isolates against metronidazole. Additionally, one *F. nucleatum* isolate (OMZ 772) and one *L. buccalis* isolate (19AF3) exceeded this value. However, varying sensitivity of *Capnocytophaga* to metronidazole has been described.^38^ The notion that susceptibilities can vary geographically was also reported by Yunoki et al,^80^ as *Fusobacterium* spp. isolates from Japanese patients showed 100% susceptibility towards metronidazole, which was therefore considered to be the most reliable antimicrobial agent against *Fusobacterium* spp. In an earlier study by Al-Ahmad et al,^2^ the MICs for *F. nucleatum* isolated from endodontic samples showed no resistance to benzylpenicillin, amoxicillin, amoxicillin-clavulanic acid, clindamycin and metronidazole. Our results are consistent with these studies, with the exception of *F. nucleatum* OMZ 772, a Swiss isolate, which yielded a MIC of >256 µg/l for metronidazole and thus clearly showed antibiotic resistance, although the nim gene was not detected. Metronidazole appears to be the antibiotic of choice in treating NG, with a protocol comprising 400 mg three times a day for three days.^19^ Because of relatively high amounts of *Fusobacterium* species (especially *F. nucleatum*) in NG patients, a potential resistance of that genus has to be taken into consideration when selecting an antibiotic.^7^

In a study in Brazil, Gomes et al^34^ examined endodontic bacteria samples and found notable resistances against benzylpenicillin and clindamycin in anaerobic bacteria, finding that mainly *F. nucleatum* strains were affected. Similarly, a study conducted in Taiwan also found high incidences of clindamycin resistance among anaerobes such as *F. nucleatum*.^66^ Yunoki et al^80^ found clindamycin alone to be a poor therapeutic agent against anaerobes. In our study, only resistant *Capnocytophaga* spp. were detected, with isolate 7AF. resistant to benzylpenicillin and isolate 21AK1 resistant to clindamycin, whereas high MICs for these two antibiotics were not found when tested on *F. nucleatum* species. The resistance was associated with the identification of the cfxA gene in both strains and with the ermF gene in the 7AF. isolate.

Anaerobic bacteria such as *F. nucleatum* are usually susceptible to fluoroquinolones. Milazzo et al^47^ reported that moxifloxacin had comparable antibacterial activity against periodontal pathogens when compared to amoxycillin-clavulanic acid and showed even better results than clindamycin, metronidazole and penicillin. In a previous study,^21^ MICs for moxifloxacin between 0.006 and 0.25 µg/l were reported for *F. nucleatum*, whereas in the present study, these values range from 0.002 to 12 µg/l. Nevertheless, other authors showed an increase in resistant bacterial isolates towards fluoroquinolones over time, with an increase of moxifloxacin resistance (MIC ≥ 4 µg/l) from 30% to 43% in anaerobic *Bacteroides* isolates in the US from 1994 to 2001.^33^

*Capnocytophaga* species were sensitive to all tested antibiotics except metronidazole, clindamycin and in one case benzylpenicillin. Beta-lactamase production should be observed, as it is the most common mechanism of resistance to beta-lactam antibiotics in anaerobes.^46^ Interestingly, a study by Baquero et al^4^ showed some degree of susceptibility to metronidazole and marked sensitivity towards clindamycin in *Capnocytophaga ochracea* isolates. Our results showed resistance to metronidazole in 5 of 12 *Capnocytophaga* isolates, with metronidazole MICs exceeding 4 µg/l.

With the exception of clindamycin, our data are consistent with the effective antibiotics listed by Jolivet-Gougeon et al.^37^ Two *Capnocytophaga* spp. isolates from China showed MICs exceeding 256 µg/l, a fact which demonstrates their resistance against clindamycin. All other MICs for *Capnocytophaga* isolates were ≤ 0.016 µg/l.

*Capnocytophaga* isolates were susceptible to penicillin antibiotics (benzylpenicillin, amoxicillin) with the exception of *Capnocytophaga* sp. 7AF2, a Chinese isolate, which showed resistance to benzylpenicillin (MIC 0.75 µg/l) and carried the cfxA gene.

All *Leptotrichia* isolates showed resistance to moxifloxacin. Resistance against moxifloxacin was also found in Korean *Leptotrichia* isolates (MIC 8-16 µg/l).^13^ An amino acid substitution in gyrA as the typical molecular basis of acquired fluoroquinolone resistance^22^ was not identified. A report by Eribe and Olsen^25^ found *Leptotrichia* to be susceptible to penicillin, clindamycin, metronidazole, tetracycline, imipenem, rifampicin and chloramphenicol, but resistant to erythromycin, gentamycin, kanamycin, vancomycin, fluoroquinolones and aminoglycosides. In summary, it appears that *L. buccalis* is sensitive to penicillin, tetracycline and clindamycin, and therefore to standard antimicrobial agents.

The excessive use of antibiotics is the one of the main causes for the development of antimicrobial resistance, and high levels of antibiotic use correlate with high levels of antibiotic resistance among bacterial species.^63^ A systematic review by Yin et al^79^ found that the proportion of antibiotic use in China is excessive, with an average of 50.3% of outpatients being prescribed antibiotics. The World Health Organization (WHO) does not recommend the proportion of antibiotic use exceed 30%.^79^ Particularly in developing countries, increasing levels of antibiotic consumption are observed, with the BRICS countries (Brazil, Russia, India, China, South Africa) account for 76% of the overall increase in global antibiotic consumption.^71^ Antibiotic stewardship aims to improve how antibiotics are prescribed and used in order to reduce the problem of antibiotic resistance, thus avoiding unnecessary medical complications and growing mortality rates. Since it can be assumed that localised antibiotic resistance can spread worldwide, it is international relevant to find the most effective way of handling antibiotics.^35^ Deaths caused by antimicrobial resistance are predicted to rise to 300 million a year by 2050, which will then be the leading cause of death worldwide.^12^

However, it is not only global prescription practices that need to be optimised, but also those of dentists. Many dentists in Europe as well as worldwide practice inadequate prophylactic and therapeutic antibiotic prescription regarding indications and regimes.^16,64^ Incorrect prescription of antibiotics not only leads to suboptimal treatment of the patient, but often manifests in over-prescribing of antibiotics, which in turn accelerates antibiotic resistance.

Bacterial samples from cases of periodontitis and NG should be cultured and the clinical isolates tested for their resistance pattern. In this study, antibiotic-resistant gram-negative oral bacteria were identified using the reliable E-test method. The E-test method for susceptibility testing of periodontal pathogens is a quick, useful and simple method that has been used in various studies.^38^ Multiple drugs can be tested and the method is convenient for testing individual isolates.^59^ Thus, the present study contributes to the growing number of studies on resistance patterns in oral bacteria.^40,41,72^ It can serve as the basis for further analysis of these bacterial species from other geographic regions or from patients with different oral diseases. Apparently effective antibiotics were shown to have lost their in-vitro activity against some of the tested bacterial isolates. Given the increasing problem of antibiotic resistance, these findings should stimulate further interest in analysing the extent and origin of these resistances.

## Conclusions

Due to the effectiveness of antibiotic therapies, the use of antibiotics in dentistry has increased, and as a result, oral bacteria with increased antibiotic resistance have emerged. The dental community generally tends to over-prescribe antibiotics, mainly for the prevention of infections such as endocarditis. Nevertheless, this study shows high in-vitro susceptibility of gram-negative anaerobes and capnophilic bacteria to amoxicillin and amoxicillin-clavulanic acid. Therefore, amoxicillin and amoxicillin-clavulanic acid could serve as alternatives for effective treatment of oral diseases. Because susceptibility profiles can vary widely, it is essential to continuously identify the susceptibility profiles of specific bacterial species to ensure successful treatment.
